# In-person sports event attendance and residents' health-related wellbeing: empirical evidence from CGSS 2023

**DOI:** 10.3389/fpubh.2026.1906946

**Published:** 2026-07-30

**Authors:** Minmin Wei, Qing Wei, Zixia Wang

**Affiliations:** 1 School of Physical Education, Hunan University of Technology, Zhuzhou, China; 2 Department of Physical Education and Health Teaching and Research, Hunan Applied Technology College, Changde, China

**Keywords:** CGSS 2023, health-related wellbeing, in-person sports event attendance, physical exercise, potential mediating pathway, scene theory

## Abstract

**Background:**

In-person sports event attendance is an important way for residents to encounter sporting events, engage in leisure activities, and expand social interaction. Examining the relationships among in-person sports event attendance, physical exercise, and residents' health-related wellbeing can help clarify the public health value of sports event resources.

**Methods:**

Using data from the 2023 Chinese General Social Survey (CGSS 2023), this study measured residents' health-related wellbeing across four dimensions: physical health, mental health, social interaction, and subjective happiness. Ordinary least squares (OLS) regression, potential mediating-pathway tests, ordered logit models, binary logit models, alternative-indicator tests, and propensity score matching (PSM) were used to examine the association between in-person sports event attendance and health-related wellbeing and the potential behavioral pathway involving physical exercise.

**Results:**

In-person sports event attendance was significantly and positively associated with residents' health-related wellbeing (β = 0.412, *p* < 0.01). Standardized dimension-specific results showed that the association was relatively more apparent for social interaction (β = 0.151, *p* < 0.01), followed by subjective happiness (β = 0.054, *p* < 0.01) and physical health (β = 0.037, *p* < 0.01); the association with mental health was positive and marginally significant (β = 0.025, *p* < 0.10). In-person attendance was also significantly associated with physical exercise (β = 0.419, *p* < 0.01), and physical exercise was significantly associated with health-related wellbeing (β = 0.314, *p* < 0.01). Bootstrap tests showed an indirect association through physical exercise of 0.123, accounting for 29.8% of the total association. Subgroup regressions supported the positive association across urban-rural residence, age, and education groups, but interaction tests did not show significant between-group differences.

**Conclusion:**

In-person sports event attendance is consistently and positively associated with residents' health-related wellbeing, with social interaction being the most prominent dimension. Physical exercise may constitute a potential mediating pathway linking in-person attendance and health-related wellbeing. Sports event resources should be connected with community health promotion and public sports services, and support mechanisms should be strengthened to link viewing experiences with everyday physical activity.

## Introduction

Sports participation is an important way for residents to maintain health, enrich leisure life, and support quality of life. Existing research on sports and health has mainly focused on active behaviors such as physical exercise, physical activity, and sport participation. A large body of evidence suggests that regular physical exercise is closely related to better physical functioning, better mental status, and higher life satisfaction (1–7). However, residents' engagement with sports is not limited to direct participation. With the development of professional sports, mass sporting events, and offline event provision, in-person sports event attendance has gradually become an important form through which residents experience sporting events, participate in sports consumption, and expand social interaction (8). Compared with television, internet, and mobile-platform viewing, in-person attendance emphasizes bodily presence, situated experience, and real-time interaction. Compared with physical exercise, in-person attendance highlights event experience, emotional involvement, and social interaction, and may be associated with residents' health-related wellbeing through emotional arousal, identity, social connection, and the awakening of interest in sports.

Existing studies provide an important basis for understanding the public health value of sports viewing. Research has found positive associations between sports viewing and outcomes such as self-rated health, life satisfaction, subjective wellbeing, and depressive symptoms (9–12). However, the relationships among different viewing modes, event types, and health outcomes are not fully consistent. Compared with television, internet, and mobile-platform viewing, in-person attendance places greater emphasis on bodily presence, shared experience, and interpersonal interaction, and its relationship with health-related wellbeing may have distinctive features. In the Chinese context, research has begun to examine the relationships between sports viewing and residents' happiness, social capital, and social interaction (13–16). Overall, however, two issues remain underdeveloped. First, health outcomes have often been concentrated on single indicators, such as subjective happiness or depressive symptoms, with insufficient attention to the comprehensive and multidimensional nature of health-related wellbeing. Second, the potential behavioral pathways linking sports viewing and health-related wellbeing remain insufficiently explained, particularly the possible role of physical exercise.

Based on these gaps, this study uses CGSS 2023 (17) data to examine the relationship between in-person sports event attendance and residents' health-related wellbeing and to further analyze the potential behavioral pathway involving physical exercise. The theoretical and empirical contributions of this study are mainly threefold. First, drawing on Scene Theory, this study conceptualizes in-person attendance as a public health-promoting setting composed of sports facilities, event activities, spectator interaction, and sports culture, and further understands it as a form of indirect sports participation distinct from direct physical exercise, thereby providing a theoretical explanation for why in-person attendance may be related to health-related wellbeing. Second, physical exercise is placed within the behavioral chain through which setting experience may be transformed into health outcomes, and the study examines whether it constitutes a potential behavioral pathway linking in-person attendance and health-related wellbeing. Third, health-related wellbeing is characterized across four dimensions: physical health, mental health, social interaction, and subjective happiness, and the analysis combines both a composite indicator and dimension-specific outcomes, thereby extending the dominant single-outcome framework in previous studies. The findings deepen understanding of the public health value of sports viewing and provide empirical reference for linking sports event resources with community-based national fitness and health-promotion services.

## Literature review and hypotheses

Scene Theory and the health-promoting logic of in-person sports event attendance

Scene Theory was systematically developed by Clark and colleagues in urban sociology and was further elaborated in later work on scenescapes. Its core idea is that concrete spaces can be understood as meaning systems jointly constituted by material facilities, activity practices, participating groups, and cultural values (18, 19). A scene is therefore not merely a physical location; rather, it is a social space that can influence individual experience and behavioral choices through environmental atmosphere, interactive relationships, and value expression. For this study, Scene Theory is not used to provide a general explanation of urban development. Instead, it offers a more focused framework for understanding why the site of a sports event may become a public health-promoting setting that shapes emotional experience, social interaction, identity, and behavioral motivation.

Accordingly, in-person sports event attendance can be understood as a form of indirect sports participation that differs from direct physical exercise. Although spectators do not directly compete or exercise, their bodily presence, shared viewing, interactive cheering, and sport identification embed them in a setting jointly constituted by venues, event activities, spectator groups, and sports culture. This setting may be associated with health-related wellbeing through two pathways: first, a direct association through emotional arousal, social connection, and identity; and second, an indirect association through the stimulation of sport interest and exercise motivation by athletic display, role-model effects, and peer interaction, which may extend the viewing experience into everyday physical exercise. Existing research suggests that urban scenes, as cultural meaning systems, can influence residents' life happiness (20), and studies of exercise for health promotion in setting-based frameworks also emphasize the importance of environmental support, behavioral motivation, and social support for health behavior (21). On this basis, this study organizes its hypotheses around the logic of the “in-person attendance setting-physical exercise behavior-health-related wellbeing” pathway.

### In-person sports event attendance and residents' health-related wellbeing

Within the above Scene Theory framework, in-person sports event attendance is not only a leisure-entertainment and sports-consumption behavior but also a form of indirect sports participation embedded in concrete sport-event settings. Compared with mediated viewing through television, the internet, or mobile platforms, in-person attendance emphasizes bodily presence, venue atmosphere, and real-time interaction, while also being shaped by venue accessibility, event supply, and transportation conditions. Therefore, in-person attendance may be associated with residents' health-related wellbeing through setting experience, emotional involvement, and social interaction.

Existing research provides empirical support for understanding the relationship between in-person attendance and health-related wellbeing. Kawakami et al. (11), based on longitudinal data from Japanese adults, found that watching sports events was favorably associated with subsequent healthy lifestyles, mental health, and happiness. Tsuji et al. (12) further reported that both in-person attendance and television/internet viewing were associated with a lower risk of depressive symptoms and higher social wellbeing, especially in indicators of social connection such as participation in sports groups and meeting friends. In the Chinese context, Wei and Zhao (13), using CGSS data, found that watching sports events was positively associated with residents' subjective wellbeing and that physical health and social capital may constitute related pathways. These studies indicate that sports viewing is not merely passive recreation; rather, it may be linked to individual health status, psychological experience, and social relationships.

From the perspective of process, in-person attendance may be related to health-related wellbeing through emotional experience and social connection. On the one hand, the competitive process, uncertainty of outcomes, and athletes' performances during sporting events may evoke enjoyment, excitement, identification, and positive emotions among spectators. Studies have found that watching professional baseball is associated with older adults' affective experience, subjective happiness, and health-related outcomes (22, 23), and sports viewing may also be related to subjective vitality through enjoyment, reward mechanisms, and team identification (9, 10). On the other hand, sporting events have strong public-topic and collective-situation attributes. Spectators can communicate around game results, athlete performances, and team identification, forming social connections through shared viewing, discussion, and cheering. Chinese studies have also found positive relationships between watching sports events and subjective wellbeing, physical health, social capital, and social interaction (14–16).

Health-related wellbeing is not equivalent to a single dimension of physical health or short-term pleasure. It includes physical condition, psychological feelings, social relationships, and life evaluation. wellbeing research has suggested that wellbeing in exercise and sport settings includes not only hedonic experience but also bodily vitality, meaning, autonomy, and overall life status (24–26). Given the available CGSS 2023 items and the focus of this study, we characterize residents' health-related wellbeing through four dimensions: physical health, mental health, social interaction, and subjective happiness. It should also be noted that the relationship between in-person attendance and wellbeing does not occur outside social structures; such associations may be constrained by economic capital, cultural capital, social capital, and regional sport-resource conditions (27). Therefore, the empirical analysis controls for demographic and socioeconomic variables and further examines the relationship between in-person attendance and different dimensions of health-related wellbeing.

Hypothesis 1: In-person sports event attendance is significantly and positively associated with residents' health-related wellbeing.

Considering the multidimensional structure of health-related wellbeing, in-person attendance may be associated with physical health, mental health, social interaction, and subjective happiness to different degrees. The following dimension-specific hypotheses are therefore proposed:

Hypothesis 1a: In-person sports event attendance is significantly and positively associated with residents' physical health.

Hypothesis 1b: In-person sports event attendance is significantly and positively associated with residents' mental health.

Hypothesis 1c: In-person sports event attendance is significantly and positively associated with residents' social interaction.

Hypothesis 1d: In-person sports event attendance is significantly and positively associated with residents' subjective happiness.

### In-person sports event attendance and physical exercise

In-person sports event attendance may also be related to residents' physical exercise behavior. Sporting events display athletic skills, competitive processes, body images, and sporting spirit, and may strengthen residents' interest in and identification with sports activities, creating demonstration, motivational, and interest-awakening links. When residents form positive evaluations of a sport, athlete, or team through attendance, the viewing experience may be associated with subsequent willingness to participate in sport, thereby forming a potential behavioral pathway from sport exposure to physical exercise.

Existing studies provide empirical support for the potential link from viewing to participation. Kawakami et al. (11) found that watching sports events was associated with a lower risk of physical inactivity and that in-person attendance was favorably associated with readiness for health behavior change. Aral and Nicolaides (28) showed that exercise behavior may spread through social networks, which provides insight into how sports viewing may be linked to sport participation through peer interaction, group identification, and social diffusion. Evidence from sports social media research also points to a similar idea: when sport-related health communication is embedded in interaction and support, it may help users form more positive attitudes toward sport participation (29). Although Tsuji et al. (12) did not find a clear positive association between sports viewing and health behavior among older adults, their study showed that sports viewing was associated with sports-group participation and meeting friends. This suggests that viewing itself may not automatically correspond to sufficient physical activity, but it may create conditions for later physical exercise through sport exposure and social connection.

From the perspectives of social identity and behavioral motivation, role models and group atmosphere in sporting events may be associated with a stronger perceived value of exercise. Yoshida et al. (10) showed that sports consumption is not an isolated viewing behavior but is intertwined with identity, attendance, and positive psychological experience. Therefore, in-person attendance may be positively associated with residents' exercise frequency through sport interest, exercise identity, and positive emotional experience.

At the same time, the relationship between in-person attendance and physical exercise is not inevitable. Some attendance may remain at the level of static watching and may be accompanied by sedentary behavior. Hamer et al. (30) suggested that televised sports viewing may be associated with physical inactivity and obesity risk. Kawakami et al. (11) also noted that viewing form, frequency, and health outcomes may differ. Thus, in-person attendance should not be assumed to translate automatically into actual exercise. It is more appropriate to understand it as a potential antecedent that may stimulate sport interest and willingness to participate. Based on this reasoning, the following hypothesis is proposed:

Hypothesis 2: In-person sports event attendance is significantly and positively associated with residents' physical exercise.

### Physical exercise and health-related wellbeing

Physical exercise is an important behavioral factor related to residents' health-related wellbeing. Compared with in-person attendance, physical exercise involves more direct bodily practice and may be associated with wellbeing through better physical functioning, emotional regulation, a stronger sense of control, and broader social interaction. Existing studies have shown that physical exercise is closely related to subjective wellbeing, social capital, hope, peer relationships, social support, exercise identity, and health consciousness, and these factors may play mediating or moderating roles in the relationship between exercise and wellbeing (1–3). From a broader wellbeing perspective, exercise behavior may bring short-term enjoyment and may also be related to bodily vitality, life meaning, and overall quality of life (24, 25).

Specifically, physical exercise may be associated with wellbeing at physical, psychological, and social levels. Kekalainen et al. (4) found both cross-sectional and longitudinal associations between leisure-time physical activity, mental wellbeing, and subjective health, and different types of activity may correspond to different dimensions of wellbeing benefits. Ruthig et al. (31) showed that a higher sense of control was associated with better psychological and physical wellbeing among older adults, suggesting that physical exercise may be linked to individuals' health status and sense of control through perceptible and sustainable bodily action. Physical exercise may also be related to subjective wellbeing through basic psychological need satisfaction, sleep quality, positive emotional experience, mindfulness, and emotion regulation (5–7).

In addition, physical exercise has a social-interaction attribute. Community fitness, group exercise, and shared sports activities with friends may provide residents with social-interaction settings and increase emotional support and social identity. Therefore, the relationship between physical exercise and health-related wellbeing is not limited to physical health; it may also be associated with better physical condition, psychological experience, and social relationships. Based on this reasoning, the following hypothesis is proposed:

Hypothesis 3: Physical exercise is significantly and positively associated with residents' health-related wellbeing.

### Physical exercise as a potential mediating pathway

In summary, the theoretical logic of this study can be summarized as follows: as a public health-promoting setting, in-person sports event attendance may be directly associated with health-related wellbeing through event experience, emotional involvement, social interaction, and identity. Meanwhile, athletic display, role-model effects, and peer interaction in the event setting may stimulate sport interest and exercise motivation, making physical exercise a potential behavioral pathway linking in-person attendance and health-related wellbeing. In other words, this study does not only examine whether attendance is beneficial; it further explores how the attendance setting may be connected with health-related wellbeing through the behavioral pathway of physical exercise. Based on this reasoning, the following hypothesis is proposed:

Hypothesis 4: Physical exercise may serve as a potential behavioral pathway and play a mediating role between in-person sports event attendance and residents' health-related wellbeing.

## Materials and methods

### Data source and sample selection

This study uses data from the 2023 Chinese General Social Survey (CGSS 2023) (17). The CGSS is a nationwide comprehensive social survey conducted by the National Survey Research Center at Renmin University of China. It covers demographic characteristics, socioeconomic status, lifestyle, health status, and social attitudes and therefore provides a suitable data basis for examining the relationships among in-person sports event attendance, physical exercise, and residents' health-related wellbeing. Based on the research focus, this study retained items related to in-person sports event attendance, physical exercise, physical health, mental health, social interaction, subjective happiness, and relevant demographic and socioeconomic control variables. Responses such as “don't know,” “refusal,” “not applicable,” and other invalid values were treated as missing. Cases with missing values on core variables or control variables were then deleted, yielding a final analytic sample of 5,411 valid observations.

### Measures

Dependent variable: health-related wellbeing. Health-related wellbeing is comprehensive and multidimensional, including residents' physical health status, psychological feelings, social relationships, and quality of life. Previous studies have selected relevant indicators according to research questions and data availability. Huang et al. (32), in the context of sports participation and health-related wellbeing, emphasized the combined role of physical health, mental health, and social interaction. Yang et al. (33) operationalized older adults' health-related wellbeing through dimensions such as self-rated health, life satisfaction, and depressive symptoms. Zhang et al. (34) argued that health-related wellbeing concerns not only individual health status but also quality of life and people's happiness. Drawing on these studies, the theoretical analysis above, and the item structure of CGSS 2023, this study measures residents' health-related wellbeing through four dimensions: physical health, mental health, social interaction, and subjective happiness. It should be noted that the four dimensions are all single-item indicators in CGSS. The composite variable is used primarily as a summary indicator of overall health-related wellbeing and should not be interpreted as a strict psychometric latent construct. To reduce the potential construct-validity risk of simple summation, this study also models the four dimensions separately as dependent variables and further uses standardized composite indicators, alternative composite indicators, and indicators excluding the social interaction dimension in robustness checks.

Specifically, physical health was measured by item A15, “How would you rate your current physical health?” and coded from 1 = very unhealthy to 5 = very healthy. Mental health was measured by item A17, ‘In the past 4 weeks, how often have you felt depressed or down?' and reverse-coded as 1 = always, 2 = often, 3 = sometimes, 4 = rarely, and 5 = never; higher values indicate fewer depressive or downhearted feelings and better mental health. Social interaction was measured by item A30–7, ‘In the past year, did you often gather with friends during your leisure time?' and reverse-coded as 1 = never, 2 = several times a year or less, 3 = several times a month, 4 = several times a week, and 5 = every day; higher values indicate more frequent gatherings with friends. Subjective happiness was measured by item A36, “Overall, do you feel that your life is happy?” and coded from 1 = very unhappy to 5 = very happy. The four dimensions were summed with equal weights to generate the composite health-related wellbeing variable, with a theoretical range of 4 to 20; higher values indicate higher health-related wellbeing.

Core explanatory variable: in-person sports event attendance. This variable was derived from CGSS 2023 item A30-10, ‘In the past year, did you often watch sports events in person during your leisure time?' The original response options ranged from every day to never. To align the direction of the variable with the research meaning, the item was reverse-coded as 1 = never, 2 = several times a year or less, 3 = several times a month, 4 = several times a week, and 5 = every day; higher values indicate more frequent in-person sports event attendance. To avoid confusion with viewing through television, the internet, or mobile platforms, this variable is referred to throughout the manuscript as in-person sports event attendance.

Mediator: physical exercise. This variable was derived from CGSS 2023 item A30-9, “In the past year, did you often participate in physical exercise during your leisure time?” Consistent with the in-person attendance variable, physical exercise frequency was reverse-coded as 1 = never, 2 = several times a year or less, 3 = several times a month, 4 = several times a week, and 5 = every day; higher values indicate more frequent physical exercise.

Control variables. To reduce omitted-variable bias, this study selected age, sex, household registration, education, pension insurance, socioeconomic status, and marital status as control variables based on existing research and CGSS 2023 data availability. Age was calculated as 2023 minus the respondent's year of birth. Sex was coded as 0 = female and 1 = male. Household registration was coded as 0 = rural and 1 = urban. Education was coded as 0 = below junior high school and 1 = junior high school or above. Pension insurance was coded as 0 = no pension insurance and 1 = has pension insurance. Socioeconomic status was based on self-rated socioeconomic status and coded as 0 = middle or below and 1 = upper-middle or upper. Marital status was coded as 0 = no spouse and 1 = has spouse.

### Model specification and testing strategy

To test the research hypotheses, this study used ordinary least squares (OLS) regression as the baseline model and reported robust standard errors. Because the analysis is based on cross-sectional data, the models are used to identify statistical associations among variables rather than causal effects. The baseline regressions used a stepwise specification: a model including only control variables was first estimated, in-person sports event attendance was then added, and physical exercise was finally included to observe changes in the key coefficients. The baseline, dimension-specific, and potential mediating-pathway model specifications are presented in Equations 1–6.


$${\text{HRW}}_{\text{i}}\text{ = }\alpha \text{0 + }\theta {\text{0}}^{\prime }X_{i}\text{ + }\varepsilon_{i}$$ (1)

$${\text{HRW}}_{\text{i}}\text{= }\alpha \text{1 + }\beta {\text{1}Attend}_{\text{i}}\text{ + }\theta {\text{1}}^{\prime }X_{i}\text{ + }\varepsilon_{\text{i}}$$ (2)

$${\text{HRW}}_{\text{i}}\text{= }\alpha_{\text{2}}\text{ + }\beta_{\text{2}}{Attend}_{\text{i}}\text{ + }\beta {\text{3}Exercise}_{i}\text{ + }\theta_{2}^{'}X_{i}\text{ + }\varepsilon_{i}$$ (3)

where HRW_*i*_ denotes the health-related wellbeing of respondent *i*, Attend_*i*_ denotes in-person sports event attendance frequency, Exercise_*i*_ denotes physical exercise frequency, *X*_*i*_ denotes the set of control variables, and ε_*i*_ is the random error term. If β1 is significantly positive, in-person attendance is positively associated with health-related wellbeing. If β_2_ remains significant but becomes smaller than β1 after physical exercise is included, this may preliminarily suggest that physical exercise shows features of a potential mediating pathway.

Because health-related wellbeing is composed of physical health, mental health, social interaction, and subjective happiness, the study further estimated regressions using each dimension as the dependent variable to examine the association between in-person attendance and different dimensions:


$${\text{Y}}_{\text{ki}}\text{ = }\alpha_{\text{k}}\text{ + }\beta_{\text{k}}{\text{Attend}}_{\text{i}}\text{ + }\theta_{k}^{\prime }{\text{X}}_{\text{i}}\text{ + }\varepsilon_{\text{ki}},\text{ k = 1},\text{ 2},\text{ 3},\text{ 4}$$ (4)

where *Y*_*ki*_ represents physical health, mental health, social interaction, and subjective happiness. Because the four dimensions differ in conceptual meaning and measurement properties, this study does not directly compare the strength of dimensions based on unstandardized coefficients. Instead, standardized indicators are used in the robustness checks to further examine relative patterns.

To examine the potential mediating pathway through physical exercise, this study used the regression coefficient method and a bootstrap test with 1,000 replications to assess the indirect association. The potential mediating-pathway models are specified as follows:


$${\text{Exercise}}_{i}\text{ = }\alpha_{\text{3}}\text{ + }{aAttend}_{i}\text{ + }\theta_{3}^{1}X_{i}\text{ + }\mu_{\text{i}}$$ (5)

$${\text{HRW}}_{i}\text{= }\alpha_{\text{4}}\text{ + }c^{\prime }{Attend}_{i}\text{ + }b{Exercise}_{i}\text{ + }\theta_{4}^{'}X_{i}\text{ + }\nu_{\text{i}}$$ (6)

where *a* × *b* represents the indirect association between in-person attendance and health-related wellbeing through physical exercise, and c′ represents the direct association after controlling for physical exercise. If the bootstrap confidence interval does not include zero, the indirect association is statistically significant, although it should still be interpreted as potential pathway evidence under cross-sectional data.

In addition, three types of robustness checks were conducted. First, health-related wellbeing was treated as an ordered variable and estimated using ordered logit models; a binary high-health-related wellbeing variable was also constructed using the median as the cutoff and estimated using logit models. Second, standardized composite indicators, alternative indicators excluding the social interaction dimension, and standardized alternative indicators were used to test whether the results were stable across different measurement strategies. Third, propensity score matching (PSM) was used as a supplementary test. Respondents with in-person attendance were treated as the treatment group, and those who never attended in person were treated as the control group. Propensity scores were estimated based on age, sex, household registration, education, pension insurance, socioeconomic status, and marital status. For heterogeneity analysis, the study conducted subgroup regressions by urban-rural residence, age, and education and further constructed interaction terms to test whether between-group differences were statistically significant.

## Results

### Descriptive statistics and correlation analysis

The average age of the sample was 54.501 years. Men accounted for 45.19% of the sample and women for 54.81%. Urban household registration accounted for 39.99%, and rural household registration accounted for 60.01%. Respondents with junior high school education or above accounted for 65.59%. Respondents participating in pension insurance accounted for 76.46%. Those reporting upper-middle or upper socioeconomic status accounted for 5.66%, and those with a spouse accounted for 74.22%. Descriptive statistics for the main variables are shown in Table 1.

**Table 1:** Descriptive statistics for the main variables.

Variable	Mean	SD	Min	Max
In-person sports event attendance	1.293	0.660	1	5
Physical exercise	2.576	1.618	1	5
Physical health	3.349	1.138	1	5
Mental health	3.847	1.123	1	5
Social interaction	2.257	1.028	1	5
Subjective happiness	3.894	0.848	1	5
Health-related wellbeing	13.348	2.742	4	20
Age	54.501	16.153	18	94
Sex	0.452	0.498	0	1
Household registration	0.400	0.490	0	1
Education	0.656	0.475	0	1
Pension insurance	0.765	0.424	0	1
Socioeconomic status	0.057	0.231	0	1
Marital status	0.742	0.437	0	1

Correlation analysis showed that in-person sports event attendance, physical exercise, and health-related wellbeing were all significantly and positively correlated. Specifically, in-person attendance was significantly correlated with physical exercise (*r* = 0.231, *p* < 0.01), indicating that more frequent in-person attendance was associated with more frequent physical exercise. In-person attendance was also significantly correlated with health-related wellbeing (*r* = 0.191, *p* < 0.01), preliminarily suggesting that attendance may be associated with higher health-related wellbeing. Physical exercise was likewise significantly correlated with health-related wellbeing (*r* = 0.272, *p* < 0.01), indicating a positive association between exercise frequency and residents' health-related wellbeing. These results provide an initial basis for further examining the potential association pathway of in-person attendance-physical exercise-health-related wellbeing.

### Relationship between in-person sports event attendance and health-related wellbeing

Table 2 presents the composite-indicator regressions and dimension-specific regressions. Model 1 includes only control variables, Model 2 adds in-person sports event attendance, and Model 3 further adds physical exercise. The results show that in-person attendance is significantly and positively associated with the composite health-related wellbeing indicator. After physical exercise is added, the coefficient decreases but remains significant, suggesting that in-person attendance has a direct association with health-related wellbeing and may also be indirectly related through physical exercise. Models 4–7 use physical health, mental health, social interaction, and subjective happiness as dependent variables. The results show that in-person attendance is significantly and positively associated with physical health, social interaction, and subjective happiness, and is positively but marginally significantly associated with mental health. Because the four dimensions differ in measurement meaning and distribution, the unstandardized coefficients should not be directly compared; relative dimensional patterns are further examined using standardized regression results.

**Table 2:** In-person sports event attendance and health-related wellbeing and its dimensions.

Variable	Model 1 health-related wellbeing	Model 2 health-related wellbeing	Model 3 health-related wellbeing	Model 4 physical health	Model 5 mental health	Model 6 social interaction	Model 7 subjective happiness
In-person sports event attendance	–	0.412^***^ (0.053)	0.290^***^ (0.052)	0.064^***^ (0.021)	0.043^*^ (0.022)	0.235^***^ (0.022)	0.069^***^ (0.017)
Physical exercise	–	–	0.293^***^ (0.023)	–	–	–	–
Age	−0.042^***^ (0.002)	−0.039^***^ (0.002)	−0.039^***^ (0.002)	−0.021^***^ (0.001)	−0.003^**^ (0.001)	−0.015^***^ (0.001)	0.000 (0.001)
Sex	0.264^***^ (0.069)	0.215^***^ (0.069)	0.178^***^ (0.068)	0.063^**^ (0.029)	0.137^***^ (0.030)	0.048^*^ (0.026)	−0.033 (0.023)
Household registration	0.709^***^ (0.076)	0.652^***^ (0.076)	0.460^***^ (0.076)	0.156^***^ (0.031)	0.197^***^ (0.033)	0.237^***^ (0.029)	0.062^**^ (0.025)
Education	1.002^***^ (0.091)	0.955^***^ (0.091)	0.788^***^ (0.091)	0.259^***^ (0.038)	0.365^***^ (0.038)	0.205^***^ (0.033)	0.126^***^ (0.030)
Pension insurance	0.107 (0.084)	0.112 (0.084)	0.087 (0.083)	0.033 (0.035)	−0.020 (0.036)	0.025 (0.032)	0.075^***^ (0.029)
Socioeconomic status	0.915^***^ (0.152)	0.870^***^ (0.152)	0.794^***^ (0.150)	0.227^***^ (0.066)	0.135^**^ (0.062)	0.173^***^ (0.059)	0.335^***^ (0.047)
Marital status	0.109 (0.082)	0.122 (0.082)	0.147^*^ (0.081)	0.053 (0.034)	0.095^***^ (0.035)	−0.129^***^ (0.031)	0.103^***^ (0.028)
Constant	14.357^***^ (0.157)	13.742^***^ (0.176)	13.355^***^ (0.176)	4.094^***^ (0.074)	3.495^***^ (0.077)	2.613^***^ (0.068)	3.540^***^ (0.061)
*N*	5,411	5,411	5,411	5,411	5,411	5,411	5,411
*R* ^2^	0.156	0.165	0.190	0.142	0.054	0.157	0.027

Robust standard errors are shown in parentheses. ^***^, ^**^, and ^*^ indicate significance at the 1%, 5%, and 10% levels, respectively. – indicates that the variable was not included.

The standardized dimension-specific regressions show that the standardized coefficient is relatively higher for social interaction (β = 0.151, *p* < 0.01), followed by subjective happiness (β = 0.054, *p* < 0.01) and physical health (β = 0.037, *p* < 0.01). The association with mental health is positive and marginally significant (β = 0.025, *p* = 0.053). This suggests that social interaction may be a relatively prominent dimension in the association between in-person attendance and health-related wellbeing.

### Potential mediating pathway through physical exercise

Table 3 reports the regression results for the potential mediating pathway through physical exercise. Model 8 shows that in-person attendance is significantly and positively associated with physical exercise (β = 0.419, *p* < 0.01), suggesting that attendance may be related to residents' exercise behavior through demonstration, interest awakening, and exercise identity; Hypothesis 2 is supported. Model 9 shows that physical exercise is significantly and positively associated with health-related wellbeing (β = 0.314, *p* < 0.01), supporting Hypothesis 3. Model 10 includes both in-person attendance and physical exercise. Both variables remain significantly and positively associated with health-related wellbeing, and the coefficient of in-person attendance decreases from 0.412 to 0.290, indicating that physical exercise may show features of a potential mediating pathway.

**Table 3:** Regression results for the potential mediating pathway through physical exercise.

Variable	Model 8 physical exercise	Model 9 health-related wellbeing	Model 10 health-related wellbeing
In-person sports event attendance	0.419^***^ (0.029)	–	0.290^***^ (0.052)
Physical exercise	–	0.314^***^ (0.022)	0.293^***^ (0.023)
Age	0.000 (0.001)	−0.041^***^ (0.002)	−0.039^***^ (0.002)
Sex	0.125^***^ (0.041)	0.209^***^ (0.068)	0.178^***^ (0.068)
Household registration	0.655^***^ (0.047)	0.485^***^ (0.076)	0.460^***^ (0.076)
Education	0.573^***^ (0.053)	0.807^***^ (0.091)	0.788^***^ (0.091)
Pension insurance	0.087^*^ (0.048)	0.081 (0.083)	0.087 (0.083)
Socioeconomic status	0.259^***^ (0.097)	0.819^***^ (0.150)	0.794^***^ (0.150)
Marital status	−0.084^*^ (0.047)	0.139^*^ (0.081)	0.147^*^ (0.081)
Constant	1.321^***^ (0.103)	13.745^***^ (0.161)	13.355^***^ (0.176)
*N*	5,411	5,411	5,411
*R* ^2^	0.146	0.186	0.190

Robust standard errors are shown in parentheses. ^***^, ^**^, and ^*^ indicate significance at the 1%, 5%, and 10% levels, respectively. – indicates that the variable was not included.

**Figure 1:**
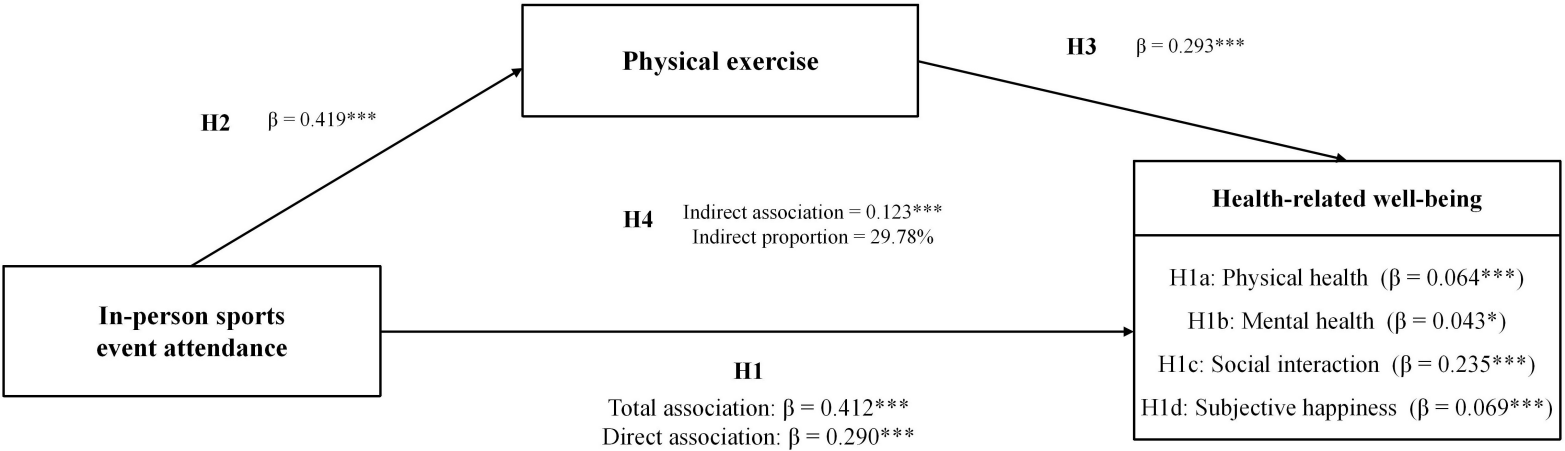
Empirical mediating-pathway diagram linking in-person sports event attendance, physical exercise, and health-related wellbeing. Coefficients shown in the figure are unstandardized regression coefficients. ***p < 0.01.

A bootstrap test with 1,000 replications was then conducted to examine the indirect association, and the results are reported in Table 4. The results show that the indirect association through physical exercise is 0.123, with a 95% confidence interval of [0.097, 0.148], which does not include zero. The direct association is 0.290, and the total association is 0.412, both significant at the 1% level. The indirect association accounts for approximately 29.8% of the total association. These results suggest that about three-tenths of the statistical association between in-person attendance and residents' health-related wellbeing is related to the physical exercise pathway, while the remaining association may be related to emotional enjoyment, social connection, identity, and other direct or indirect pathways. Thus, the hypothesis that physical exercise serves as a potential mediating pathway is statistically supported, but the result remains association-based evidence under cross-sectional data. The empirical pathway is summarized in Figure 1.

**Table 4:** Bootstrap test of the potential mediating pathway through physical exercise.

Association	Coefficient	SE	*Z*	*P*	95% CI	Proportion
Total association	0.412	0.054	7.67	*p* < 0.01	[0.307, 0.518]	100.00%
Direct association	0.290	0.053	5.46	*p* < 0.01	[0.186, 0.394]	70.22%
Indirect association	0.123	0.013	9.43	*p* < 0.01	[0.097, 0.148]	29.78%

Bootstrap resampling was repeated 1,000 times. Confidence intervals are bootstrap normal–approximation confidence intervals. The proportion is calculated as the corresponding association divided by the total association.

### Robustness checks

To examine the robustness of the main conclusions, this study conducted supplementary tests from three perspectives: model specification, indicator construction, and sample selection bias. First, model specifications and dependent-variable treatments were changed. Table 5 reports the ordered logit and binary logit estimates. The ordered logit results show that in-person attendance remains significantly and positively associated with health-related wellbeing. After physical exercise is added, both in-person attendance and physical exercise remain significantly positive. The binary logit results likewise show that in-person attendance is significantly associated with a higher probability of entering the high-health-related wellbeing group; after physical exercise is added, both variables remain significant. These results indicate that the main conclusions are consistent after changing model specifications and dependent-variable treatments.

**Table 5:** Robustness checks with ordered logit and logit models.

Variable	Ordered logit model 11	Ordered logit model 12	Logit model 13	Logit model 14
In-person sports event attendance	0.307^***^ (0.042)	0.220^***^ (0.043)	0.253^***^ (0.051)	0.165^***^ (0.051)
Physical exercise	–	0.208^***^ (0.016)	–	0.206^***^ (0.019)
Control variables	Yes	Yes	Yes	Yes
*N*	5,411	5,411	5,411	5,411
Pseudo *R*^2^	0.037	0.044	0.092	0.107

^***^indicates statistical significance at the 1% level (*p* < 0.01). Robust standard errors are shown in parentheses; – indicates that the variable was not included.

Second, the construction of the health-related wellbeing indicator was changed. Table 6 reports results using the standardized composite indicator, the alternative indicator excluding social interaction, and the standardized alternative indicator. The results show that the coefficients of in-person attendance remain positive and generally significant under different indicator treatments. After the social interaction dimension is excluded, the positive association between in-person attendance and health-related wellbeing remains, although the coefficient decreases. This suggests that the association is not driven entirely by the social interaction dimension and also indicates that social interaction is an important dimension in the relationship between in-person attendance and health-related wellbeing.

**Table 6:** Indicator-construction robustness checks.

Variable	Standardized composite model 15	Standardized composite model 16	Excluding social interaction model 17	Excluding social interaction model 18	Standardized alternative model 19	Standardized alternative model 20
In-person sports event attendance	0.101^***^ (0.013)	0.072^***^ (0.013)	0.177^***^ (0.044)	0.088^**^ (0.044)	0.059^***^ (0.014)	0.030^**^ (0.014)
Physical exercise	–	0.071^***^ (0.005)	–	0.212^***^ (0.020)	–	0.068^***^ (0.006)
Control variables	Yes	Yes	Yes	Yes	Yes	Yes
*N*	5,411	5,411	5,411	5,411	5,411	5,411
*R* ^2^	0.158	0.184	0.101	0.120	0.094	0.113

^**^ and ^***^ indicate statistical significance at the 5% and 1% levels, respectively.

Third, PSM was used as a supplementary robustness check. To reduce self-selection bias resulting from observable differences between residents with and without in-person attendance, this study further dichotomized the attendance variable. Respondents who had never attended sporting events in person were defined as the control group, and those with any in-person attendance were defined as the treatment group. Propensity scores were estimated based on age, sex, household registration, education, pension insurance, socioeconomic status, and marital status. After matching, the treatment and control groups each included 1,140 observations. Except for a small number of variables, the standardized differences in the main covariates decreased substantially, suggesting that matching strengthened group comparability to some extent. As shown in Table 7, the ATET estimate for in-person attendance is 0.653 and significant at the 1% level, indicating that after observable selection differences are controlled, residents with in-person attendance still report significantly higher health-related wellbeing scores than those without in-person attendance. The alternative indicator excluding social interaction remains significant (ATET = 0.334, *p* < 0.01), and the four dimension-specific results are generally positive. These findings are consistent with the baseline regressions and alternative-indicator tests, supporting the robustness of the positive association between in-person attendance and residents' health-related wellbeing. It should be noted that PSM can only reduce selection bias caused by observable variables and cannot fully rule out unobservable confounding or reverse causality; therefore, the PSM results should be understood as robustness evidence rather than causal identification.

**Table 7:** Propensity score matching robustness checks.

Dependent variable	ATET	SE	*Z*	*P*	95% CI	*N*
Health-related wellbeing	0.653^***^	0.098	6.65	*p* < 0.001	[0.461, 0.845]	5,411
Health-related wellbeing excluding social interaction	0.334^***^	0.085	3.95	*p* < 0.001	[0.168, 0.500]	5,411
Physical health	0.127^***^	0.041	3.11	*p* < 0.01	[0.047, 0.207]	5,411
Mental health	0.091^**^	0.043	2.12	*p* < 0.05	[0.007, 0.175]	5,411
Social interaction	0.319^***^	0.038	8.49	*p* < 0.001	[0.245, 0.392]	5,411
Subjective happiness	0.116^***^	0.034	3.42	*p* < 0.01	[0.050, 0.183]	5,411

Standard errors in the PSM results are Abadie-Imbens robust standard errors. The treatment variable is whether the respondent attended sports events in person; the estimator is ATET. ^**^ and ^***^ indicate statistical significance at the 5% and 1% levels, respectively.

### Heterogeneity analysis

To examine whether the association between in-person attendance and health-related wellbeing differs across groups, this study conducted subgroup regressions by urban-rural residence, age, and education and further tested between-group differences using interaction models. Table 8 shows that the coefficient of in-person attendance is positive and statistically significant in all subgroups. Specifically, the coefficients are 0.288 for rural residents and 0.296 for urban residents, 0.241 for those below age 60 and 0.353 for those aged 60 or above, and 0.355 for those below junior high school education and 0.268 for those with junior high school education or above. These results indicate that the positive association between in-person attendance and health-related wellbeing is supported across different population groups. However, interaction tests show that the between-group differences by urban-rural residence, age, and education are not statistically significant. Therefore, this study does not interpret the subgroup coefficient differences as strict group heterogeneity, but rather as evidence of the stability of the main conclusion across different groups.

**Table 8:** Heterogeneity analysis.

Variable	Rural	Urban	Below 60	60 and above	Below junior high	Junior high and above
In-person sports event attendance	0.288^***^ (0.075)	0.296^***^ (0.073)	0.241^***^ (0.064)	0.353^***^ (0.090)	0.355^***^ (0.133)	0.268^***^ (0.057)
Control variables	Yes	Yes	Yes	Yes	Yes	Yes
*N*	3,247	2,164	3,161	2,250	1,862	3,549
*R* ^2^	0.188	0.130	0.200	0.135	0.053	0.147
*p*-value for between-group difference	0.281	0.281	0.298	0.298	0.812	0.812

The *P*-values for between-group differences by urban-rural residence, age, and education are from interaction-term models. ^***^indicates statistical significance at the 1% level (*p* < 0.01).

## Discussion

### Main findings and interpretation

First, in-person sports event attendance is significantly and positively associated with residents' health-related wellbeing. This result supports the theoretical judgment that in-person attendance can be understood as a public health-promoting setting. In-person attendance is not simply an act of event viewing or sports consumption; rather, it is a setting experience jointly constituted by sports venues, event activities, spectator interaction, and sports culture. Although in-person attendance is not characterized primarily by direct physical activity, the emotional involvement, shared viewing, on-site interaction, and sport identification embedded in it may create conditions for residents to obtain positive emotions, expand social connections, and develop sport interest. Therefore, in-person attendance is better understood as a potential setting resource that links sports event resources, residents' leisure life, and public health-promotion services. This finding is broadly consistent with Kawakami et al. (11), who found that in-person attendance was related to healthy lifestyles, mental health, and happiness, and with Tsuji et al. (12), who reported that sports viewing was related to higher social wellbeing among older adults. Unlike studies focusing mainly on specific events, older adults, or sports fans, this study uses a general resident sample and further shows that ordinary residents' in-person attendance is positively associated with health-related wellbeing.

Second, the relationships between in-person attendance and different dimensions of health-related wellbeing vary. Standardized dimension-specific results show that the association is relatively more apparent for social interaction (β = 0.151, *p* < 0.01), followed by subjective happiness (β = 0.054, *p* < 0.01) and physical health (β = 0.037, *p* < 0.01), while the association with mental health is positive and marginally significant (β = 0.025, *p* < 0.10). This suggests that the connection between in-person attendance and health-related wellbeing may be reflected more strongly in social interaction and public-life participation than in direct mental-health differences. Sporting events have strong public-topic and collective-situation attributes. Residents may communicate around game results, athlete performances, and team support, forming shared experience, emotional resonance, and group identity, which may be associated with higher levels of social interaction. This is consistent with Tsuji et al. (12), who found that sports viewing was related to social wellbeing indicators such as meeting friends and participating in sports groups. By contrast, the relatively weaker association with mental health may be related to the fact that mental health is shaped by family relationships, economic pressure, social support, and individual emotional characteristics. It may also be related to the limited measurement of mental health in this study, which is represented only by the reverse-coded frequency of feeling depressed or down.

Third, physical exercise may show features of a potential mediating pathway between in-person attendance and residents‘ health-related wellbeing. This suggests that attendance may be associated with residents' health-related wellbeing through a pathway of interest awakening, exercise identity, and behavioral connection. The athletic performances, sporting spirit, and rules displayed during sporting events may be related to residents‘ understanding of sports, interest, and willingness to exercise. When the emotional experience and sport identification generated by attendance are connected with actual exercise behavior, in-person attendance may become an entry point for residents' sport participation. This finding is consistent with Kawakami et al. (11), who suggested that sports viewing may be related to health behavior and readiness for behavior change. It should be noted that physical exercise accounts for only part of the total association, indicating that in-person attendance may also be related to health-related wellbeing through other pathways, such as positive emotion, social support, identity, and sense of life meaning. The dimension-specific regressions and standardized dimension-specific results both suggest that social interaction is an important dimension of the association between in-person attendance and health-related wellbeing. Meanwhile, the alternative-indicator tests and PSM results show that after the social interaction dimension is excluded, the positive association remains but weakens, indicating that social interaction is an important but not exclusive source of the association.

Fourth, the positive association between in-person attendance and health-related wellbeing is relatively stable across different population groups. Subgroup regressions show that the coefficients of in-person attendance are positive and statistically significant across urban-rural residence, age, and education groups, indicating that the association is not limited to a particular group. Further interaction tests show that between-group differences by urban-rural residence, age, and education are not statistically significant. Therefore, this study does not interpret coefficient differences across groups as strict group heterogeneity, but rather as evidence of the robustness of the main conclusion across different groups.

### Theoretical contributions

The theoretical contributions of this study are mainly reflected in three aspects. First, this study introduces Scene Theory and scenescape research into research on in-person sports events and health-related wellbeing, showing that in-person attendance is not simply a viewing behavior but a public health-promoting setting jointly constituted by sports facilities, event activities, spectator interaction, and cultural values. This provides an integrated explanatory framework for mechanisms such as emotional experience, social interaction, and identity. Second, this study conceptualizes in-person attendance as indirect sports participation, extending the dominant understanding of sport participation in sport-health research that has mainly focused on direct physical exercise and emphasizing that spectator-based sport participation may also have health-related value. Third, this study explicitly places physical exercise within the theoretical chain of ‘setting experience-behavioral transformation-health-related wellbeing,' revealing that physical exercise may serve as a potential behavioral pathway linking in-person attendance and health-related wellbeing, thereby further connecting sports event research, sport participation research, and public health research.

## Limitations

This study has several limitations. First, it uses CGSS 2023 cross-sectional data. Although control variables, robustness checks, alternative-indicator tests, and PSM strengthen the reliability of the findings, the analysis cannot identify temporal order or causal direction and cannot fully rule out reverse causality or omitted-variable bias. Therefore, physical exercise should be understood as a potential behavioral pathway between in-person attendance and health-related wellbeing rather than as a confirmed causal mechanism. Future research could use longitudinal data, quasi-experimental designs, or instrumental-variable approaches for further testing. Second, the health-related wellbeing indicator is formed by equally summing four single-item dimensions: physical health, mental health, social interaction, and subjective happiness. Due to the structure of CGSS data, this approach may involve construct-validity limitations. In particular, mental health is represented only by the reverse-coded frequency of feeling depressed or down, and the measurement scope is relatively limited. Although this study adds standardized composite indicators, alternative indicators excluding social interaction, and PSM tests, future studies could use multi-item scales and objective health indicators for further validation. Third, in-person attendance, physical exercise, and social interaction are all self-reported leisure activity items from the same survey context and may involve same-source bias, common item-set bias, or a general leisure orientation that inflates associations. Fourth, because of the limitations of CGSS items, this study can only examine the overall frequency of in-person attendance and cannot identify specific sport projects, event levels, viewing duration, co-attendance modes, venue conditions, transportation accessibility, or fan identity. In addition, some dimension-specific models have limited explanatory power. For example, the *R*^2^ of the subjective happiness model is only 0.027, indicating that the current model explains only a small part of the variation in subjective happiness. Future research could further include family relationships, social support, life stress, community environment, and other relevant variables.

### Practical implications

First, sports event resources should be connected with community health-promotion services. In-person attendance is positively associated with residents' health-related wellbeing, and the association is relatively prominent for social interaction. On this basis, the setting value of sports events in promoting social interaction, enriching public life, and connecting residents with sport participation should be further strengthened. Event organization, venue services, community mobilization, and national fitness services should be integrated to strengthen the public-service attributes of sports event resources. In practice, this integration should connect event organization, venue operation, community services, and health-promotion knowledge within concrete service processes, so that one-off viewing experiences can be extended into sustainable health-promotion services. From an implementation perspective, such integration also requires knowledge management across event organizers, venue operators, community workers, and health-promotion professionals; evidence on innovation ambidexterity suggests that the search, absorption, and recombination of knowledge can support organizations in developing new service modes while maintaining existing operational strengths (35).

Second, support mechanisms linking viewing experiences with everyday sport participation should be strengthened. Physical exercise shows features of a potential mediating pathway between in-person attendance and health-related wellbeing, suggesting that sport interest, sport identification, and positive experience formed during attendance may create conditions for residents to participate in everyday physical activity. Community and public sports service organizations can provide scientific fitness consultation, beginner guidance, venue information, and low-intensity participation opportunities in connection with sporting events, thereby making it more convenient and accessible for residents to extend viewing experiences into everyday sport participation. Relevant international physical-activity guidelines also emphasize that accessible services and supportive environments should be used to help different population groups increase physical activity participation (36).

Third, the inclusiveness and accessibility of sports events and public sports services should be strengthened. The positive association between in-person attendance and health-related wellbeing is relatively stable across different groups, suggesting that service development should better respond to the common needs of general residents. In practice, community spatial conditions, transportation accessibility, age structure, and the local foundation of public sports services should be considered to optimize support environments for in-person attendance and subsequent physical participation and increase opportunities for social interaction and physical activity. While improving existing event supply and venue services, lightweight participation modes for older adults, less-educated groups, and community residents should also be explored. Therefore, event-service development should not stop at one-off event popularity or elite-event provision; instead, institutional arrangements should be formed in inclusiveness, accessibility, and sustained participation support. In this sense, public sports service innovation also involves a balance between exploiting existing event and venue resources and exploring low-threshold, community-oriented participation formats; innovation research has similarly emphasized that managing exploration and exploitation can support innovation performance (37).

## Conclusion

Based on Scene Theory and CGSS 2023 data, this study examined the relationships among in-person sports event attendance, physical exercise, and residents' health-related wellbeing. The study understands in-person attendance as a public health-promoting setting and a form of indirect sports participation, and further examines the potential behavioral pathway role of physical exercise. The main conclusions are as follows. First, in-person attendance is significantly and positively associated with residents' health-related wellbeing. Dimension-specific results show that the association is relatively more apparent for social interaction and is significantly positive for physical health and subjective happiness, while the association with mental health is positive and marginally significant. Second, in-person attendance is significantly and positively associated with physical exercise, and physical exercise is significantly and positively associated with health-related wellbeing, indicating a consistent positive association among in-person attendance, physical exercise, and health-related wellbeing. Third, physical exercise shows features of a potential mediating pathway between in-person attendance and residents' health-related wellbeing. Bootstrap results indicate that the indirect association through physical exercise accounts for 29.8% of the total association. Fourth, the positive association between in-person attendance and health-related wellbeing is relatively stable across groups. It is supported across urban-rural residence, age, and education groups, although the evidence is insufficient to support strict group heterogeneity.

## Data Availability

Publicly available datasets were analyzed in this study. The CGSS 2023 data can be found at: https://cgss.ruc.edu.cn/.
